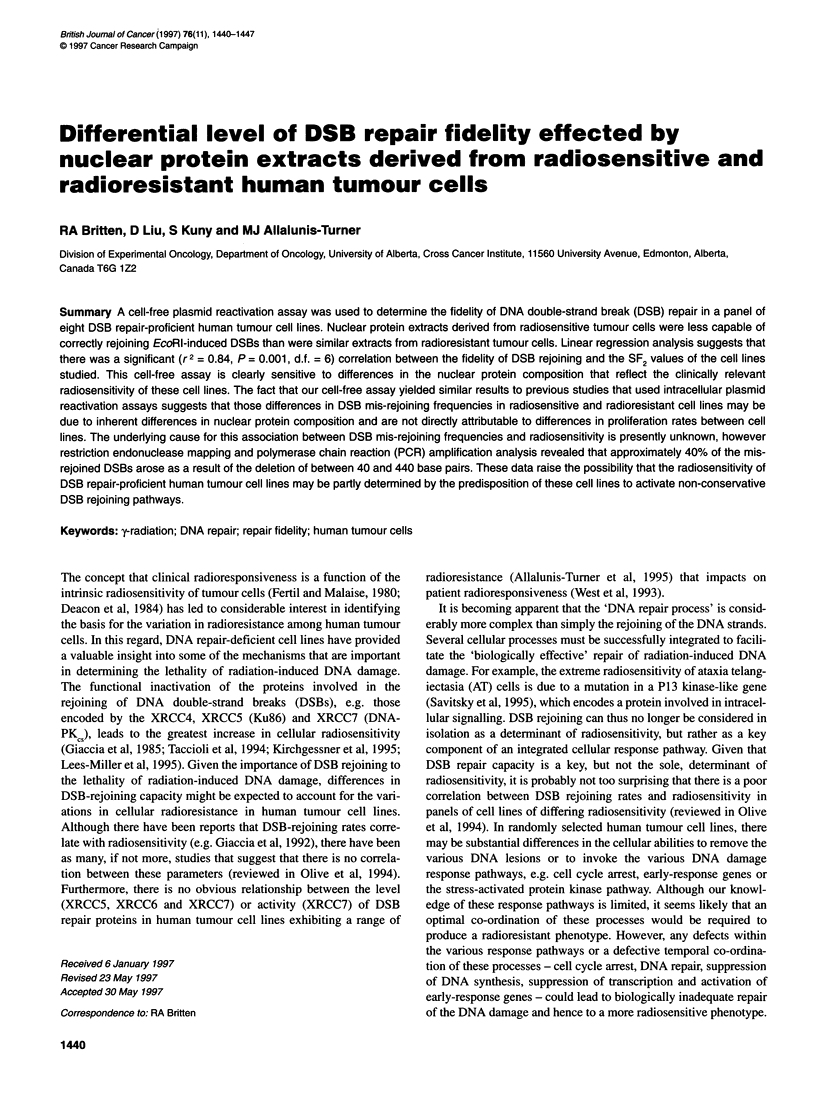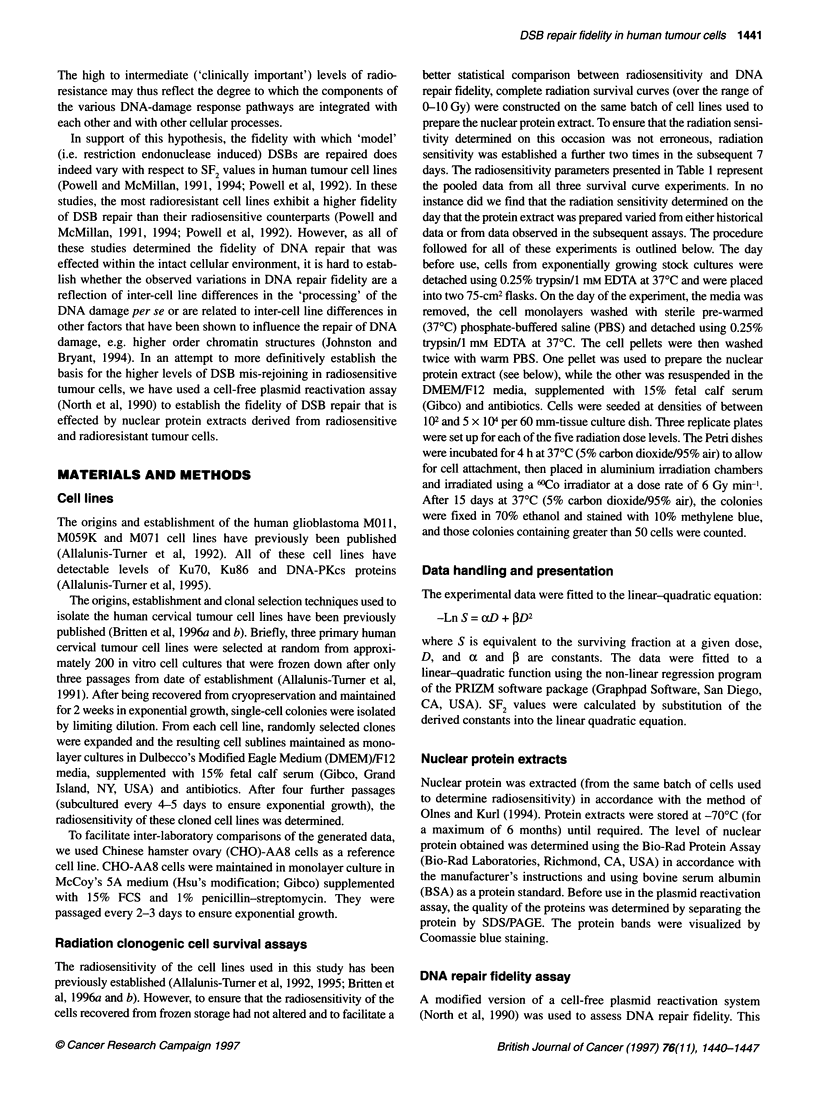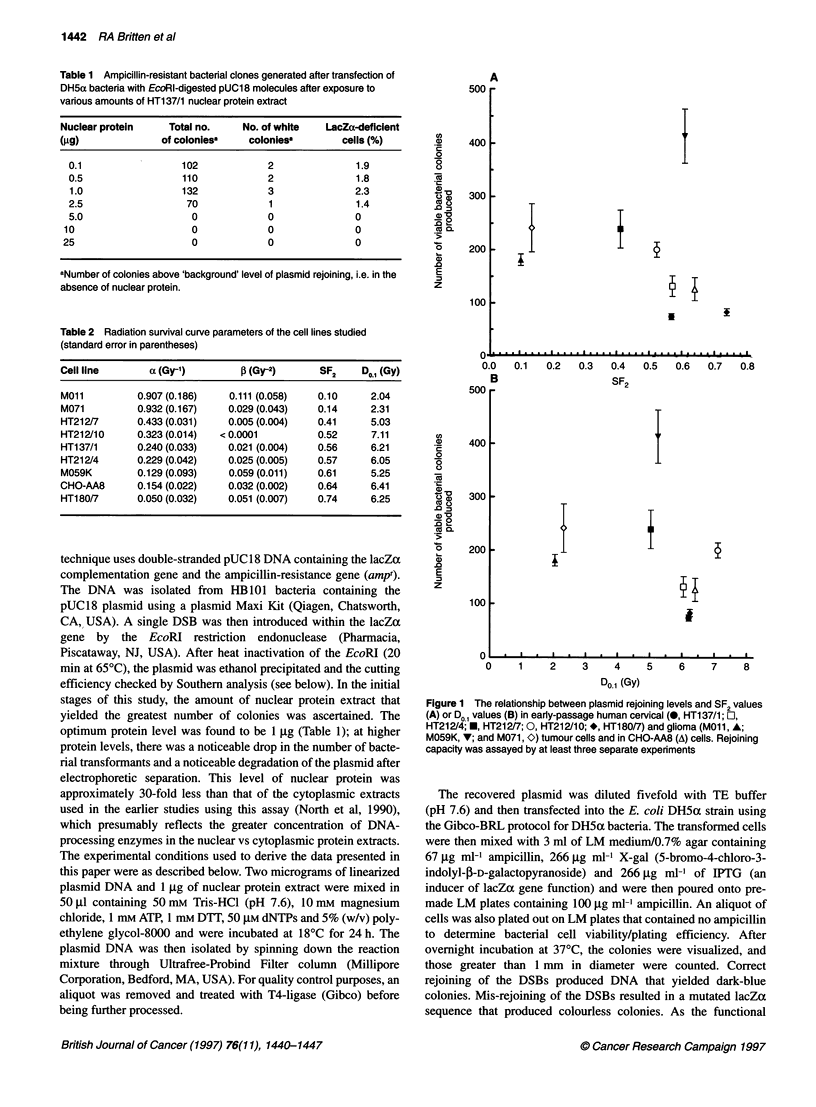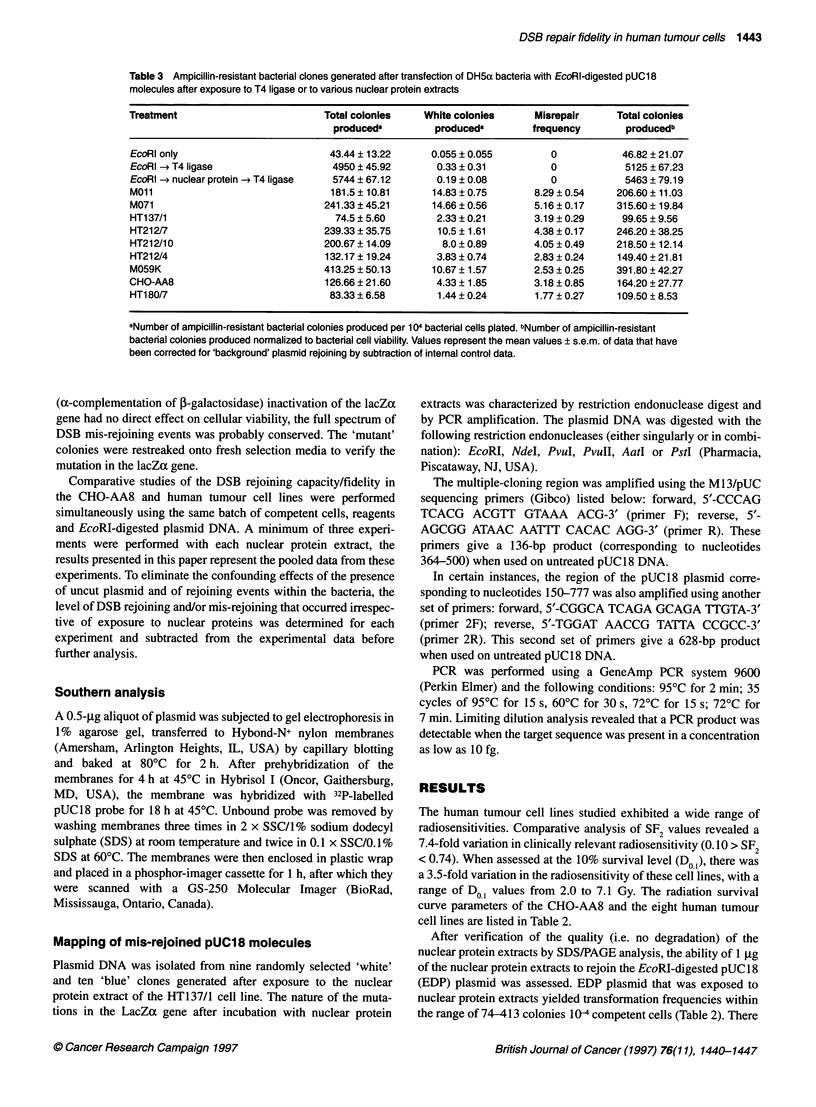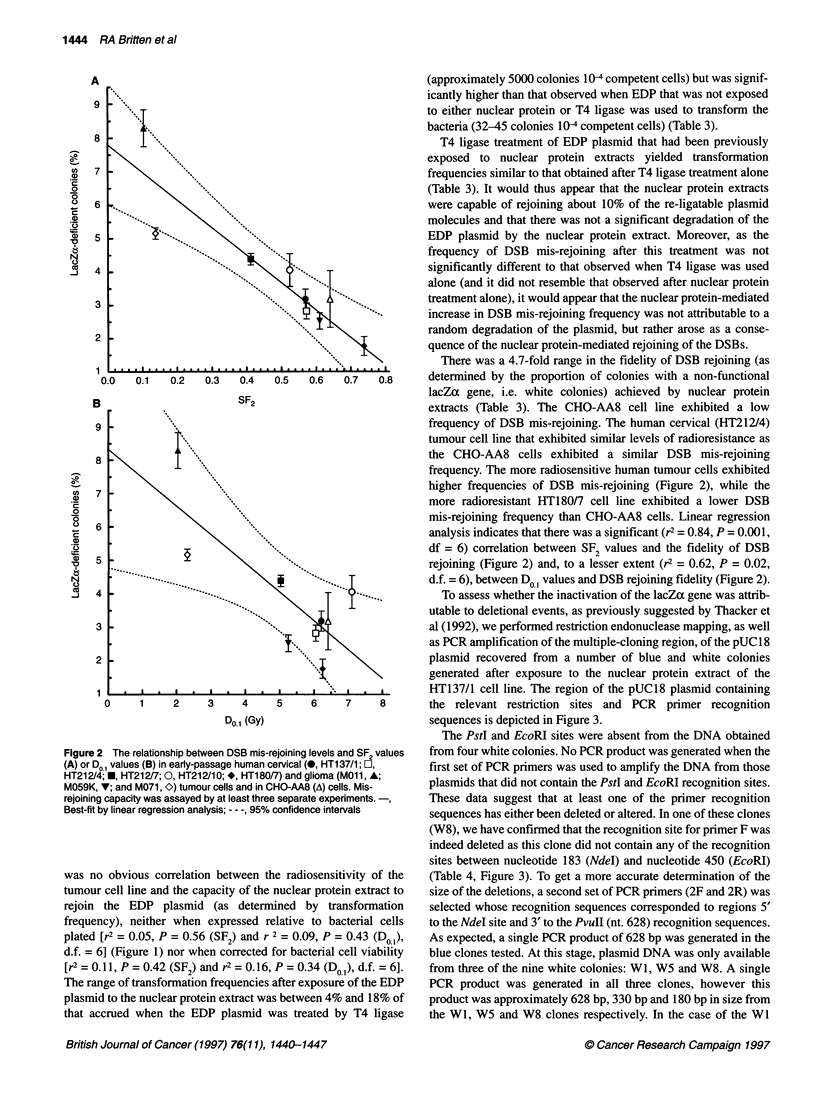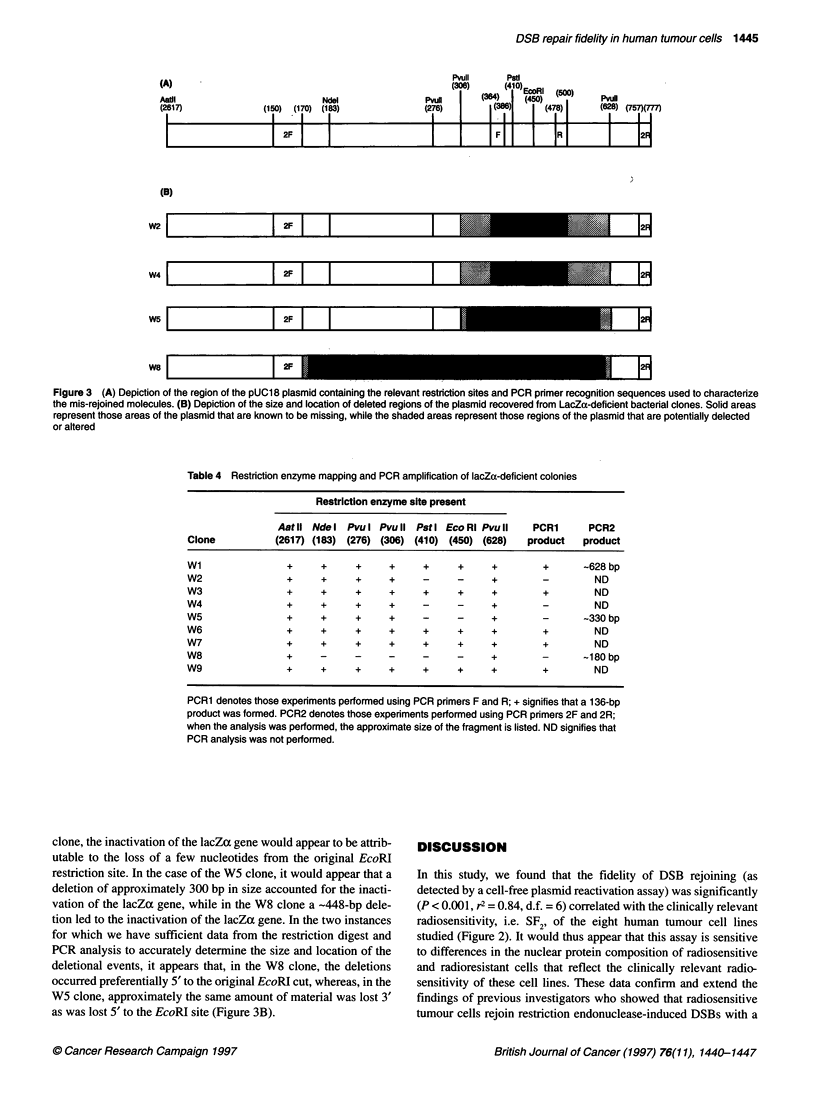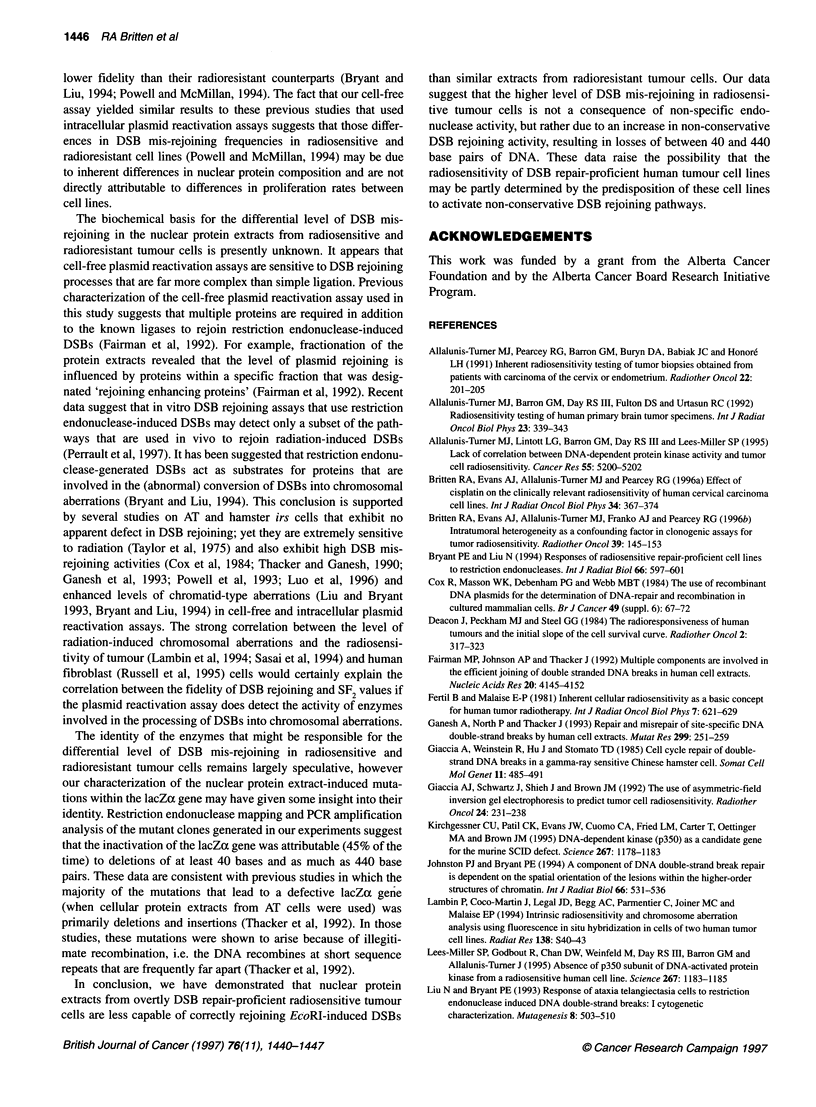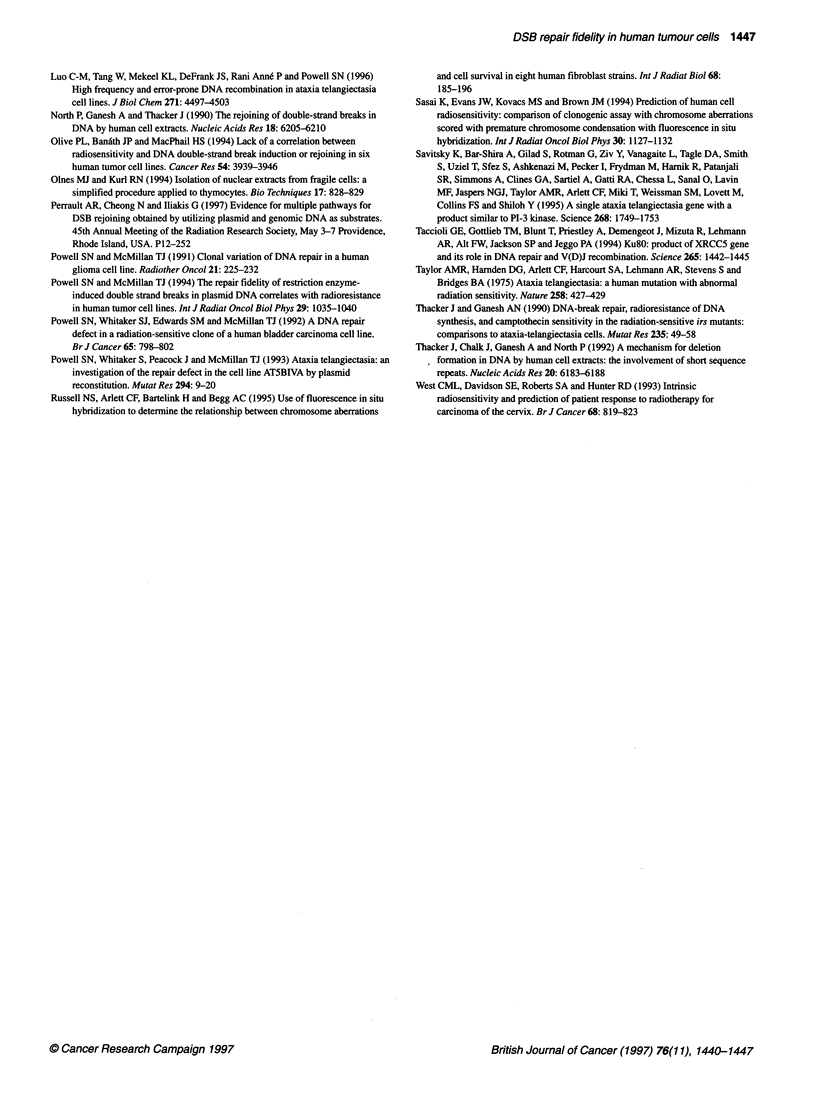# Differential level of DSB repair fidelity effected by nuclear protein extracts derived from radiosensitive and radioresistant human tumour cells.

**DOI:** 10.1038/bjc.1997.576

**Published:** 1997

**Authors:** R. A. Britten, D. Liu, S. Kuny, M. J. Allalunis-Turner

**Affiliations:** Department of Oncology, University of Alberta, Cross Cancer Institute, Edmonton, Canada.

## Abstract

A cell-free plasmid reactivation assay was used to determine the fidelity of DNA double-strand break (DSB) repair in a panel of eight DSB repair-proficient human tumour cell lines. Nuclear protein extracts derived from radiosensitive tumour cells were less capable of correctly rejoining EcoRI-induced DSBs than were similar extracts from radioresistant tumour cells. Linear regression analysis suggests that there was a significant (r2 = 0.84, P = 0.001, d.f. = 6) correlation between the fidelity of DSB rejoining and the SF2 values of the cell lines studied. This cell-free assay is clearly sensitive to differences in the nuclear protein composition that reflect the clinically relevant radiosensitivity of these cell lines. The fact that our cell-free assay yielded similar results to previous studies that used intracellular plasmid reactivation assays suggests that those differences in DSB mis-rejoining frequencies in radiosensitive and radioresistant cell lines may be due to inherent differences in nuclear protein composition and are not directly attributable to differences in proliferation rates between cell lines. The underlying cause for this association between DSB mis-rejoining frequencies and radiosensitivity is presently unknown, however restriction endonuclease mapping and polymerase chain reaction (PCR) amplification analysis revealed that approximately 40% of the mis-rejoined DSBs arose as a result of the deletion of between 40 and 440 base pairs. These data raise the possibility that the radiosensitivity of DSB repair-proficient human tumour cell lines may be partly determined by the predisposition of these cell lines to activate non-conservative DSB rejoining pathways.


British Journal of Cancer (1997) 76(11), 1440–1447
© 1997 Cancer Research Campaign

### Differential level of DSB repair fidelity effected by nuclear protein extracts derived from radiosensitive and radioresistant human tumour cells

**RA Britten, D Liu, S Kuny and MJ Allalunis-Turner**

Division of Experimental Oncology, Department of Oncology, University of Alberta, Cross Cancer Institute, 11560 University Avenue, Edmonton, Alberta, Canada T6G 1Z2

**Summary** A cell-free plasmid reactivation assay was used to determine the fidelity of DNA double-strand break (DSB) repair in a panel of eight DSB repair-proficient human tumour cell lines. Nuclear protein extracts derived from radiosensitive tumour cells were less capable of correctly rejoining *Eco*RI-induced DSBs than were similar extracts from radioresistant tumour cells. Linear regression analysis suggests that there was a significant ($r^2$ = 0.84, $P$ = 0.001, d.f. = 6) correlation between the fidelity of DSB rejoining and the $SF_2$ values of the cell lines studied. This cell-free assay is clearly sensitive to differences in the nuclear protein composition that reflect the clinically relevant radiosensitivity of these cell lines. The fact that our cell-free assay yielded similar results to previous studies that used intracellular plasmid reactivation assays suggests that those differences in DSB mis-rejoining frequencies in radiosensitive and radioresistant cell lines may be due to inherent differences in nuclear protein composition and are not directly attributable to differences in proliferation rates between cell lines. The underlying cause for this association between DSB mis-rejoining frequencies and radiosensitivity is presently unknown, however restriction endonuclease mapping and polymerase chain reaction (PCR) amplification analysis revealed that approximately 40% of the mis-rejoined DSBs arose as a result of the deletion of between 40 and 440 base pairs. These data raise the possibility that the radiosensitivity of DSB repair-proficient human tumour cell lines may be partly determined by the predisposition of these cell lines to activate non-conservative DSB rejoining pathways.

**Keywords:** γ-radiation; DNA repair; repair fidelity; human tumour cells

The concept that clinical radioresponsiveness is a function of the intrinsic radiosensitivity of tumour cells (Fertil and Malaise, 1980; Deacon et al, 1984) has led to considerable interest in identifying the basis for the variation in radioresistance among human tumour cells. In this regard, DNA repair-deficient cell lines have provided a valuable insight into some of the mechanisms that are important in determining the lethality of radiation-induced DNA damage. The functional inactivation of the proteins involved in the rejoining of DNA double-strand breaks (DSBs), e.g. those encoded by the XRCC4, XRCC5 (Ku86) and XRCC7 (DNA-$PK_{cs}$), leads to the greatest increase in cellular radiosensitivity (Giaccia et al, 1985; Taccioli et al, 1994; Kirchgessner et al, 1995; Lees-Miller et al, 1995). Given the importance of DSB rejoining to the lethality of radiation-induced DNA damage, differences in DSB-rejoining capacity might be expected to account for the variations in cellular radioresistance in human tumour cell lines. Although there have been reports that DSB-rejoining rates correlate with radiosensitivity (e.g. Giaccia et al, 1992), there have been as many, if not more, studies that suggest that there is no correlation between these parameters (reviewed in Olive et al, 1994). Furthermore, there is no obvious relationship between the level (XRCC5, XRCC6 and XRCC7) or activity (XRCC7) of DSB repair proteins in human tumour cell lines exhibiting a range of radioresistance (Allalunis-Turner et al, 1995) that impacts on patient radioresponsiveness (West et al, 1993).

*Received 6 January 1997*
*Revised 23 May 1997*
*Accepted 30 May 1997*

*Correspondence to:* RA Britten

It is becoming apparent that the ‘DNA repair process’ is considerably more complex than simply the rejoining of the DNA strands. Several cellular processes must be successfully integrated to facilitate the ‘biologically effective’ repair of radiation-induced DNA damage. For example, the extreme radiosensitivity of ataxia telangiectasia (AT) cells is due to a mutation in a P13 kinase-like gene (Savitsky et al, 1995), which encodes a protein involved in intracellular signalling. DSB rejoining can thus no longer be considered in isolation as a determinant of radiosensitivity, but rather as a key component of an integrated cellular response pathway. Given that DSB repair capacity is a key, but not the sole, determinant of radiosensitivity, it is probably not too surprising that there is a poor correlation between DSB rejoining rates and radiosensitivity in panels of cell lines of differing radiosensitivity (reviewed in Olive et al, 1994). In randomly selected human tumour cell lines, there may be substantial differences in the cellular abilities to remove the various DNA lesions or to invoke the various DNA damage response pathways, e.g. cell cycle arrest, early-response genes or the stress-activated protein kinase pathway. Although our knowledge of these response pathways is limited, it seems likely that an optimal co-ordination of these processes would be required to produce a radioresistant phenotype. However, any defects within the various response pathways or a defective temporal co-ordination of these processes – cell cycle arrest, DNA repair, suppression of DNA synthesis, suppression of transcription and activation of early-response genes – could lead to biologically inadequate repair of the DNA damage and hence to a more radiosensitive phenotype.

The high to intermediate ('clinically important') levels of radioresistance may thus reflect the degree to which the components of the various DNA-damage response pathways are integrated with each other and with other cellular processes.

In support of this hypothesis, the fidelity with which 'model' (i.e. restriction endonuclease induced) DSBs are repaired does indeed vary with respect to $SF_2$ values in human tumour cell lines (Powell and McMillan, 1991, 1994; Powell et al, 1992). In these studies, the most radioresistant cell lines exhibit a higher fidelity of DSB repair than their radiosensitive counterparts (Powell and McMillan, 1991, 1994; Powell et al, 1992). However, as all of these studies determined the fidelity of DNA repair that was effected within the intact cellular environment, it is hard to establish whether the observed variations in DNA repair fidelity are a reflection of inter-cell line differences in the 'processing' of the DNA damage *per se* or are related to inter-cell line differences in other factors that have been shown to influence the repair of DNA damage, e.g. higher order chromatin structures (Johnston and Bryant, 1994). In an attempt to more definitively establish the basis for the higher levels of DSB mis-rejoining in radiosensitive tumour cells, we have used a cell-free plasmid reactivation assay (North et al, 1990) to establish the fidelity of DSB repair that is effected by nuclear protein extracts derived from radiosensitive and radioresistant tumour cells.

### MATERIALS AND METHODS

### Cell lines

The origins and establishment of the human glioblastoma M011, M059K and M071 cell lines have previously been published (Allalunis-Turner et al, 1992). All of these cell lines have detectable levels of Ku70, Ku86 and DNA-PKcs proteins (Allalunis-Turner et al, 1995).

The origins, establishment and clonal selection techniques used to isolate the human cervical tumour cell lines have been previously published (Britten et al, 1996*a* and *b*). Briefly, three primary human cervical tumour cell lines were selected at random from approximately 200 in vitro cell cultures that were frozen down after only three passages from date of establishment (Allalunis-Turner et al, 1991). After being recovered from cryopreservation and maintained for 2 weeks in exponential growth, single-cell colonies were isolated by limiting dilution. From each cell line, randomly selected clones were expanded and the resulting cell sublines maintained as monolayer cultures in Dulbecco's Modified Eagle Medium (DMEM)/F12 media, supplemented with 15% fetal calf serum (Gibco, Grand Island, NY, USA) and antibiotics. After four further passages (subcultured every 4–5 days to ensure exponential growth), the radiosensitivity of these cloned cell lines was determined.

To facilitate inter-laboratory comparisons of the generated data, we used Chinese hamster ovary (CHO)-AA8 cells as a reference cell line. CHO-AA8 cells were maintained in monolayer culture in McCoy's 5A medium (Hsu's modification; Gibco) supplemented with 15% FCS and 1% penicillin–streptomycin. They were passaged every 2–3 days to ensure exponential growth.

### Radiation clonogenic cell survival assays

The radiosensitivity of the cell lines used in this study has been previously established (Allalunis-Turner et al, 1992, 1995; Britten et al, 1996*a* and *b*). However, to ensure that the radiosensitivity of the cells recovered from frozen storage had not altered and to facilitate a better statistical comparison between radiosensitivity and DNA repair fidelity, complete radiation survival curves (over the range of 0–10 Gy) were constructed on the same batch of cell lines used to prepare the nuclear protein extract. To ensure that the radiation sensitivity determined on this occasion was not erroneous, radiation sensitivity was established a further two times in the subsequent 7 days. The radiosensitivity parameters presented in Table 1 represent the pooled data from all three survival curve experiments. In no instance did we find that the radiation sensitivity determined on the day that the protein extract was prepared varied from either historical data or from data observed in the subsequent assays. The procedure followed for all of these experiments is outlined below. The day before use, cells from exponentially growing stock cultures were detached using 0.25% trypsin/1 mM EDTA at 37°C and were placed into two 75-$cm^2$ flasks. On the day of the experiment, the media was removed, the cell monolayers washed with sterile pre-warmed (37°C) phosphate-buffered saline (PBS) and detached using 0.25% trypsin/1 mM EDTA at 37°C. The cell pellets were then washed twice with warm PBS. One pellet was used to prepare the nuclear protein extract (see below), while the other was resuspended in the DMEM/F12 media, supplemented with 15% fetal calf serum (Gibco) and antibiotics. Cells were seeded at densities of between $10^2$ and $5 \times 10^4$ per 60 mm-tissue culture dish. Three replicate plates were set up for each of the five radiation dose levels. The Petri dishes were incubated for 4 h at 37°C (5% carbon dioxide/95% air) to allow for cell attachment, then placed in aluminium irradiation chambers and irradiated using a $^{60}Co$ irradiator at a dose rate of 6 Gy $min^{-1}$. After 15 days at 37°C (5% carbon dioxide/95% air), the colonies were fixed in 70% ethanol and stained with 10% methylene blue, and those colonies containing greater than 50 cells were counted.

### Data handling and presentation

The experimental data were fitted to the linear–quadratic equation:

$$-\text{Ln}\, S = \alpha D + \beta D^2$$

where $S$ is equivalent to the surviving fraction at a given dose, $D$, and $\alpha$ and $\beta$ are constants. The data were fitted to a linear–quadratic function using the non-linear regression program of the PRIZM software package (Graphpad Software, San Diego, CA, USA). $SF_2$ values were calculated by substitution of the derived constants into the linear quadratic equation.

### Nuclear protein extracts

Nuclear protein was extracted (from the same batch of cells used to determine radiosensitivity) in accordance with the method of Olnes and Kurl (1994). Protein extracts were stored at −70°C (for a maximum of 6 months) until required. The level of nuclear protein obtained was determined using the Bio-Rad Protein Assay (Bio-Rad Laboratories, Richmond, CA, USA) in accordance with the manufacturer's instructions and using bovine serum albumin (BSA) as a protein standard. Before use in the plasmid reactivation assay, the quality of the proteins was determined by separating the protein by SDS/PAGE. The protein bands were visualized by Coomassie blue staining.

### DNA repair fidelity assay

A modified version of a cell-free plasmid reactivation system (North et al, 1990) was used to assess DNA repair fidelity. This

© Cancer Research Campaign 1997

**Table 1** Ampicillin-resistant bacterial clones generated after transfection of DH5α bacteria with *Eco*RI-digested pUC18 molecules after exposure to various amounts of HT137/1 nuclear protein extract

| Nuclear protein (μg) | Total no. of colonies[a] | No. of white colonies[a] | LacZα-deficient cells (%) |
|---|---|---|---|
| 0.1 | 102 | 2 | 1.9 |
| 0.5 | 110 | 2 | 1.8 |
| 1.0 | 132 | 3 | 2.3 |
| 2.5 | 70 | 1 | 1.4 |
| 5.0 | 0 | 0 | 0 |
| 10 | 0 | 0 | 0 |
| 25 | 0 | 0 | 0 |

[a]Number of colonies above 'background' level of plasmid rejoining, i.e. in the absence of nuclear protein.

**Table 2** Radiation survival curve parameters of the cell lines studied (standard error in parentheses)

| Cell line | α ($Gy^{-1}$) | β ($Gy^{-2}$) | $SF_2$ | $D_{0.1}$ (Gy) |
|---|---|---|---|---|
| M011 | 0.907 (0.186) | 0.111 (0.058) | 0.10 | 2.04 |
| M071 | 0.932 (0.167) | 0.029 (0.043) | 0.14 | 2.31 |
| HT212/7 | 0.433 (0.031) | 0.005 (0.004) | 0.41 | 5.03 |
| HT212/10 | 0.323 (0.014) | < 0.0001 | 0.52 | 7.11 |
| HT137/1 | 0.240 (0.033) | 0.021 (0.004) | 0.56 | 6.21 |
| HT212/4 | 0.229 (0.042) | 0.025 (0.005) | 0.57 | 6.05 |
| M059K | 0.129 (0.093) | 0.059 (0.011) | 0.61 | 5.25 |
| CHO-AA8 | 0.154 (0.022) | 0.032 (0.002) | 0.64 | 6.41 |
| HT180/7 | 0.050 (0.032) | 0.051 (0.007) | 0.74 | 6.25 |

technique uses double-stranded pUC18 DNA containing the lacZα complementation gene and the ampicillin-resistance gene (*amp*[r]). The DNA was isolated from HB101 bacteria containing the pUC18 plasmid using a plasmid Maxi Kit (Qiagen, Chatsworth, CA, USA). A single DSB was then introduced within the lacZα gene by the *Eco*RI restriction endonuclease (Pharmacia, Piscataway, NJ, USA). After heat inactivation of the *Eco*RI (20 min at 65°C), the plasmid was ethanol precipitated and the cutting efficiency checked by Southern analysis (see below). In the initial stages of this study, the amount of nuclear protein extract that yielded the greatest number of colonies was ascertained. The optimum protein level was found to be 1 μg (Table 1); at higher protein levels, there was a noticeable drop in the number of bacterial transformants and a noticeable degradation of the plasmid after electrophoretic separation. This level of nuclear protein was approximately 30-fold less than that of the cytoplasmic extracts used in the earlier studies using this assay (North et al, 1990), which presumably reflects the greater concentration of DNA-processing enzymes in the nuclear vs cytoplasmic protein extracts. The experimental conditions used to derive the data presented in this paper were as described below. Two micrograms of linearized plasmid DNA and 1 μg of nuclear protein extract were mixed in 50 μl containing 50 mM Tris-HCl (pH 7.6), 10 mM magnesium chloride, 1 mM ATP, 1 mM DTT, 50 μM dNTPs and 5% (w/v) polyethylene glycol-8000 and were incubated at 18°C for 24 h. The plasmid DNA was then isolated by spinning down the reaction mixture through Ultrafree-Probind Filter column (Millipore Corporation, Bedford, MA, USA). For quality control purposes, an aliquot was removed and treated with T4-ligase (Gibco) before being further processed.

**Figure 1** The relationship between plasmid rejoining levels and $SF_2$ values (**A**) or $D_{0.1}$ values (**B**) in early-passage human cervical (●, HT137/1; □, HT212/4; ■, HT212/7; ○, HT212/10; ◆, HT180/7) and glioma (M011, ▲; M059K, ▼; and M071, ◇) tumour cells and in CHO-AA8 (Δ) cells. Rejoining capacity was assayed by at least three separate experiments

The recovered plasmid was diluted fivefold with TE buffer (pH 7.6) and then transfected into the *E. coli* DH5α strain using the Gibco-BRL protocol for DH5α bacteria. The transformed cells were then mixed with 3 ml of LM medium/0.7% agar containing 67 μg $ml^{-1}$ ampicillin, 266 μg $ml^{-1}$ X-gal (5-bromo-4-chloro-3-indolyl-β-D-galactopyranoside) and 266 μg $ml^{-1}$ of IPTG (an inducer of lacZα gene function) and were then poured onto pre-made LM plates containing 100 μg $ml^{-1}$ ampicillin. An aliquot of cells was also plated out on LM plates that contained no ampicillin to determine bacterial cell viability/plating efficiency. After overnight incubation at 37°C, the colonies were visualized, and those greater than 1 mm in diameter were counted. Correct rejoining of the DSBs produced DNA that yielded dark-blue colonies. Mis-rejoining of the DSBs resulted in a mutated lacZα sequence that produced colourless colonies. As the functional

© Cancer Research Campaign 1997

**Table 3** Ampicillin-resistant bacterial clones generated after transfection of DH5α bacteria with *Eco*RI-digested pUC18 molecules after exposure to T4 ligase or to various nuclear protein extracts

| Treatment | Total colonies produced[a] | White colonies produced[a] | Misrepair frequency | Total colonies produced[b] |
|---|---|---|---|---|
| *Eco*RI only | 43.44 ± 13.22 | 0.055 ± 0.055 | 0 | 46.82 ± 21.07 |
| *Eco*RI → T4 ligase | 4950 ± 45.92 | 0.33 ± 0.31 | 0 | 5125 ± 67.23 |
| *Eco*RI → nuclear protein → T4 ligase | 5744 ± 67.12 | 0.19 ± 0.08 | 0 | 5463 ± 79.19 |
| M011 | 181.5 ± 10.81 | 14.83 ± 0.75 | 8.29 ± 0.54 | 206.60 ± 11.03 |
| M071 | 241.33 ± 45.21 | 14.66 ± 0.56 | 5.16 ± 0.17 | 315.60 ± 19.84 |
| HT137/1 | 74.5 ± 5.60 | 2.33 ± 0.21 | 3.19 ± 0.29 | 99.65 ± 9.56 |
| HT212/7 | 239.33 ± 35.75 | 10.5 ± 1.61 | 4.38 ± 0.17 | 246.20 ± 38.25 |
| HT212/10 | 200.67 ± 14.09 | 8.0 ± 0.89 | 4.05 ± 0.49 | 218.50 ± 12.14 |
| HT212/4 | 132.17 ± 19.24 | 3.83 ± 0.74 | 2.83 ± 0.24 | 149.40 ± 21.81 |
| M059K | 413.25 ± 50.13 | 10.67 ± 1.57 | 2.53 ± 0.25 | 391.80 ± 42.27 |
| CHO-AA8 | 126.66 ± 21.60 | 4.33 ± 1.85 | 3.18 ± 0.85 | 164.20 ± 27.77 |
| HT180/7 | 83.33 ± 6.58 | 1.44 ± 0.24 | 1.77 ± 0.27 | 109.50 ± 8.53 |

[a]Number of ampicillin-resistant bacterial colonies produced per $10^4$ bacterial cells plated. [b]Number of ampicillin-resistant bacterial colonies produced normalized to bacterial cell viability. Values represent the mean values ± s.e.m. of data that have been corrected for 'background' plasmid rejoining by subtraction of internal control data.

(α-complementation of β-galactosidase) inactivation of the lacZα gene had no direct effect on cellular viability, the full spectrum of DSB mis-rejoining events was probably conserved. The 'mutant' colonies were restreaked onto fresh selection media to verify the mutation in the lacZα gene.

Comparative studies of the DSB rejoining capacity/fidelity in the CHO-AA8 and human tumour cell lines were performed simultaneously using the same batch of competent cells, reagents and *Eco*RI-digested plasmid DNA. A minimum of three experiments were performed with each nuclear protein extract, the results presented in this paper represent the pooled data from these experiments. To eliminate the confounding effects of the presence of uncut plasmid and of rejoining events within the bacteria, the level of DSB rejoining and/or mis-rejoining that occurred irrespective of exposure to nuclear proteins was determined for each experiment and subtracted from the experimental data before further analysis.

### Southern analysis

A 0.5-μg aliquot of plasmid was subjected to gel electrophoresis in 1% agarose gel, transferred to Hybond-N+ nylon membranes (Amersham, Arlington Heights, IL, USA) by capillary blotting and baked at 80°C for 2 h. After prehybridization of the membranes for 4 h at 45°C in Hybrisol I (Oncor, Gaithersburg, MD, USA), the membrane was hybridized with $^{32}P$-labelled pUC18 probe for 18 h at 45°C. Unbound probe was removed by washing membranes three times in 2 × SSC/1% sodium dodecyl sulphate (SDS) at room temperature and twice in 0.1 × SSC/0.1% SDS at 60°C. The membranes were then enclosed in plastic wrap and placed in a phosphor-imager cassette for 1 h, after which they were scanned with a GS-250 Molecular Imager (BioRad, Mississauga, Ontario, Canada).

### Mapping of mis-rejoined pUC18 molecules

Plasmid DNA was isolated from nine randomly selected 'white' and ten 'blue' clones generated after exposure to the nuclear protein extract of the HT137/1 cell line. The nature of the mutations in the LacZα gene after incubation with nuclear protein extracts was characterized by restriction endonuclease digest and by PCR amplification. The plasmid DNA was digested with the following restriction endonucleases (either singularly or in combination): *Eco*RI, *Nde*I, *Pvu*I, *Pvu*II, *Aat*I or *Pst*I (Pharmacia, Piscataway, NJ, USA).

The multiple-cloning region was amplified using the M13/pUC sequencing primers (Gibco) listed below: forward, 5′-CCCAG TCACG ACGTT GTAAA ACG-3′ (primer F); reverse, 5′-AGCGG ATAAC AATTT CACAC AGG-3′ (primer R). These primers give a 136-bp product (corresponding to nucleotides 364–500) when used on untreated pUC18 DNA.

In certain instances, the region of the pUC18 plasmid corresponding to nucleotides 150–777 was also amplified using another set of primers: forward, 5′-CGGCA TCAGA GCAGA TTGTA-3′ (primer 2F); reverse, 5′-TGGAT AACCG TATTA CCGCC-3′ (primer 2R). This second set of primers give a 628-bp product when used on untreated pUC18 DNA.

PCR was performed using a GeneAmp PCR system 9600 (Perkin Elmer) and the following conditions: 95°C for 2 min; 35 cycles of 95°C for 15 s, 60°C for 30 s, 72°C for 15 s; 72°C for 7 min. Limiting dilution analysis revealed that a PCR product was detectable when the target sequence was present in a concentration as low as 10 fg.

### RESULTS

The human tumour cell lines studied exhibited a wide range of radiosensitivities. Comparative analysis of $SF_2$ values revealed a 7.4-fold variation in clinically relevant radiosensitivity (0.10 > $SF_2$ < 0.74). When assessed at the 10% survival level ($D_{0.1}$), there was a 3.5-fold variation in the radiosensitivity of these cell lines, with a range of $D_{0.1}$ values from 2.0 to 7.1 Gy. The radiation survival curve parameters of the CHO-AA8 and the eight human tumour cell lines are listed in Table 2.

After verification of the quality (i.e. no degradation) of the nuclear protein extracts by SDS/PAGE analysis, the ability of 1 μg of the nuclear protein extracts to rejoin the *Eco*RI-digested pUC18 (EDP) plasmid was assessed. EDP plasmid that was exposed to nuclear protein extracts yielded transformation frequencies within the range of 74–413 colonies $10^{-4}$ competent cells (Table 2). There

© Cancer Research Campaign 1997

**Figure 2** The relationship between DSB mis-rejoining levels and $SF_2$ values (**A**) or $D_{0.1}$ values (**B**) in early-passage human cervical (●, HT137/1; □, HT212/4; ■, HT212/7; ○, HT212/10; ◆, HT180/7) and glioma (M011, ▲; M059K, ▼; and M071, ◇) tumour cells and in CHO-AA8 (Δ) cells. Mis-rejoining capacity was assayed by at least three separate experiments. —, Best-fit by linear regression analysis; - - -, 95% confidence intervals was no obvious correlation between the radiosensitivity of the tumour cell line and the capacity of the nuclear protein extract to rejoin the EDP plasmid (as determined by transformation frequency), neither when expressed relative to bacterial cells plated [$r^2$ = 0.05, $P$ = 0.56 ($SF_2$) and $r^2$ = 0.09, $P$ = 0.43 ($D_{0.1}$), d.f. = 6] (Figure 1) nor when corrected for bacterial cell viability [$r^2$ = 0.11, $P$ = 0.42 ($SF_2$) and $r^2$ = 0.16, $P$ = 0.34 ($D_{0.1}$), d.f. = 6]. The range of transformation frequencies after exposure of the EDP plasmid to the nuclear protein extract was between 4% and 18% of that accrued when the EDP plasmid was treated by T4 ligase (approximately 5000 colonies $10^{-4}$ competent cells) but was significantly higher than that observed when EDP that was not exposed to either nuclear protein or T4 ligase was used to transform the bacteria (32–45 colonies $10^{-4}$ competent cells) (Table 3).

T4 ligase treatment of EDP plasmid that had been previously exposed to nuclear protein extracts yielded transformation frequencies similar to that obtained after T4 ligase treatment alone (Table 3). It would thus appear that the nuclear protein extracts were capable of rejoining about 10% of the re-ligatable plasmid molecules and that there was not a significant degradation of the EDP plasmid by the nuclear protein extract. Moreover, as the frequency of DSB mis-rejoining after this treatment was not significantly different to that observed when T4 ligase was used alone (and it did not resemble that observed after nuclear protein treatment alone), it would appear that the nuclear protein-mediated increase in DSB mis-rejoining frequency was not attributable to a random degradation of the plasmid, but rather arose as a consequence of the nuclear protein-mediated rejoining of the DSBs.

There was a 4.7-fold range in the fidelity of DSB rejoining (as determined by the proportion of colonies with a non-functional lacZα gene, i.e. white colonies) achieved by nuclear protein extracts (Table 3). The CHO-AA8 cell line exhibited a low frequency of DSB mis-rejoining. The human cervical (HT212/4) tumour cell line that exhibited similar levels of radioresistance as the CHO-AA8 cells exhibited a similar DSB mis-rejoining frequency. The more radiosensitive human tumour cells exhibited higher frequencies of DSB mis-rejoining (Figure 2), while the more radioresistant HT180/7 cell line exhibited a lower DSB mis-rejoining frequency than CHO-AA8 cells. Linear regression analysis indicates that there was a significant ($r^2$ = 0.84, $P$ = 0.001, df = 6) correlation between $SF_2$ values and the fidelity of DSB rejoining (Figure 2) and, to a lesser extent ($r^2$ = 0.62, $P$ = 0.02, d.f. = 6), between $D_{0.1}$ values and DSB rejoining fidelity (Figure 2).

To assess whether the inactivation of the lacZα gene was attributable to deletional events, as previously suggested by Thacker et al (1992), we performed restriction endonuclease mapping, as well as PCR amplification of the multiple-cloning region, of the pUC18 plasmid recovered from a number of blue and white colonies generated after exposure to the nuclear protein extract of the HT137/1 cell line. The region of the pUC18 plasmid containing the relevant restriction sites and PCR primer recognition sequences is depicted in Figure 3.

The *Pst*I and *Eco*RI sites were absent from the DNA obtained from four white colonies. No PCR product was generated when the first set of PCR primers was used to amplify the DNA from those plasmids that did not contain the *Pst*I and *Eco*RI recognition sites. These data suggest that at least one of the primer recognition sequences has either been deleted or altered. In one of these clones (W8), we have confirmed that the recognition site for primer F was indeed deleted as this clone did not contain any of the recognition sites between nucleotide 183 (*Nde*I) and nucleotide 450 (*Eco*RI) (Table 4, Figure 3). To get a more accurate determination of the size of the deletions, a second set of PCR primers (2F and 2R) was selected whose recognition sequences corresponded to regions 5′ to the *Nde*I site and 3′ to the *Pvu*II (nt. 628) recognition sequences. As expected, a single PCR product of 628 bp was generated in the blue clones tested. At this stage, plasmid DNA was only available from three of the nine white colonies: W1, W5 and W8. A single PCR product was generated in all three clones, however this product was approximately 628 bp, 330 bp and 180 bp in size from the W1, W5 and W8 clones respectively. In the case of the W1

© Cancer Research Campaign 1997

**Figure 3** (**A**) Depiction of the region of the pUC18 plasmid containing the relevant restriction sites and PCR primer recognition sequences used to characterize the mis-rejoined molecules. (**B**) Depiction of the size and location of deleted regions of the plasmid recovered from LacZα-deficient bacterial clones. Solid areas represent those areas of the plasmid that are known to be missing, while the shaded areas represent those regions of the plasmid that are potentially delected or altered

**Table 4** Restriction enzyme mapping and PCR amplification of lacZα-deficient colonies

| | Restriction enzyme site present | | | | | | | | |
|---|---|---|---|---|---|---|---|---|---|
| **Clone** | ***Aat* II (2617)** | ***Nde* I (183)** | ***Pvu* I (276)** | ***Pvu* II (306)** | ***Pst* I (410)** | ***Eco* RI (450)** | ***Pvu* II (628)** | **PCR1 product** | **PCR2 product** |
| W1 | + | + | + | + | + | + | + | + | ~628 bp |
| W2 | + | + | + | + | – | – | + | – | ND |
| W3 | + | + | + | + | + | + | + | + | ND |
| W4 | + | + | + | + | – | – | + | – | ND |
| W5 | + | + | + | + | – | – | + | – | ~330 bp |
| W6 | + | + | + | + | + | + | + | + | ND |
| W7 | + | + | + | + | + | + | + | + | ND |
| W8 | + | – | – | – | – | – | + | – | ~180 bp |
| W9 | + | + | + | + | + | + | + | + | ND |

PCR1 denotes those experiments performed using PCR primers F and R; + signifies that a 136-bp product was formed. PCR2 denotes those experiments performed using PCR primers 2F and 2R; when the analysis was performed, the approximate size of the fragment is listed. ND signifies that PCR analysis was not performed.

clone, the inactivation of the lacZα gene would appear to be attributable to the loss of a few nucleotides from the original *Eco*RI restriction site. In the case of the W5 clone, it would appear that a deletion of approximately 300 bp in size accounted for the inactivation of the lacZα gene, while in the W8 clone a ~448-bp deletion led to the inactivation of the lacZα gene. In the two instances for which we have sufficient data from the restriction digest and PCR analysis to accurately determine the size and location of the deletional events, it appears that, in the W8 clone, the deletions occurred preferentially 5′ to the original *Eco*RI cut, whereas, in the W5 clone, approximately the same amount of material was lost 3′ as was lost 5′ to the *Eco*RI site (Figure 3B).

### DISCUSSION

In this study, we found that the fidelity of DSB rejoining (as detected by a cell-free plasmid reactivation assay) was significantly ($P < 0.001$, $r^2 = 0.84$, d.f. = 6) correlated with the clinically relevant radiosensitivity, i.e. $SF_2$, of the eight human tumour cell lines studied (Figure 2). It would thus appear that this assay is sensitive to differences in the nuclear protein composition of radiosensitive and radioresistant cells that reflect the clinically relevant radiosensitivity of these cell lines. These data confirm and extend the findings of previous investigators who showed that radiosensitive tumour cells rejoin restriction endonuclease-induced DSBs with a

© Cancer Research Campaign 1997

lower fidelity than their radioresistant counterparts (Bryant and Liu, 1994; Powell and McMillan, 1994). The fact that our cell-free assay yielded similar results to these previous studies that used intracellular plasmid reactivation assays suggests that those differences in DSB mis-rejoining frequencies in radiosensitive and radioresistant cell lines (Powell and McMillan, 1994) may be due to inherent differences in nuclear protein composition and are not directly attributable to differences in proliferation rates between cell lines.

The biochemical basis for the differential level of DSB mis-rejoining in the nuclear protein extracts from radiosensitive and radioresistant tumour cells is presently unknown. It appears that cell-free plasmid reactivation assays are sensitive to DSB rejoining processes that are far more complex than simple ligation. Previous characterization of the cell-free plasmid reactivation assay used in this study suggests that multiple proteins are required in addition to the known ligases to rejoin restriction endonuclease-induced DSBs (Fairman et al, 1992). For example, fractionation of the protein extracts revealed that the level of plasmid rejoining is influenced by proteins within a specific fraction that was designated 'rejoining enhancing proteins' (Fairman et al, 1992). Recent data suggest that in vitro DSB rejoining assays that use restriction endonuclease-induced DSBs may detect only a subset of the pathways that are used in vivo to rejoin radiation-induced DSBs (Perrault et al, 1997). It has been suggested that restriction endonuclease-generated DSBs act as substrates for proteins that are involved in the (abnormal) conversion of DSBs into chromosomal aberrations (Bryant and Liu, 1994). This conclusion is supported by several studies on AT and hamster *irs* cells that exhibit no apparent defect in DSB rejoining; yet they are extremely sensitive to radiation (Taylor et al, 1975) and also exhibit high DSB mis-rejoining activities (Cox et al, 1984; Thacker and Ganesh, 1990; Ganesh et al, 1993; Powell et al, 1993; Luo et al, 1996) and enhanced levels of chromatid-type aberrations (Liu and Bryant 1993, Bryant and Liu, 1994) in cell-free and intracellular plasmid reactivation assays. The strong correlation between the level of radiation-induced chromosomal aberrations and the radiosensitivity of tumour (Lambin et al, 1994; Sasai et al, 1994) and human fibroblast (Russell et al, 1995) cells would certainly explain the correlation between the fidelity of DSB rejoining and $SF_2$ values if the plasmid reactivation assay does detect the activity of enzymes involved in the processing of DSBs into chromosomal aberrations.

The identity of the enzymes that might be responsible for the differential level of DSB mis-rejoining in radiosensitive and radioresistant tumour cells remains largely speculative, however our characterization of the nuclear protein extract-induced mutations within the lacZα gene may have given some insight into their identity. Restriction endonuclease mapping and PCR amplification analysis of the mutant clones generated in our experiments suggest that the inactivation of the lacZα gene was attributable (45% of the time) to deletions of at least 40 bases and as much as 440 base pairs. These data are consistent with previous studies in which the majority of the mutations that lead to a defective lacZα gene (when cellular protein extracts from AT cells were used) was primarily deletions and insertions (Thacker et al, 1992). In those studies, these mutations were shown to arise because of illegitimate recombination, i.e. the DNA recombines at short sequence repeats that are frequently far apart (Thacker et al, 1992).

In conclusion, we have demonstrated that nuclear protein extracts from overtly DSB repair-proficient radiosensitive tumour cells are less capable of correctly rejoining *Eco*RI-induced DSBs than similar extracts from radioresistant tumour cells. Our data suggest that the higher level of DSB mis-rejoining in radiosensitive tumour cells is not a consequence of non-specific endonuclease activity, but rather due to an increase in non-conservative DSB rejoining activity, resulting in losses of between 40 and 440 base pairs of DNA. These data raise the possibility that the radiosensitivity of DSB repair-proficient human tumour cell lines may be partly determined by the predisposition of these cell lines to activate non-conservative DSB rejoining pathways.

### ACKNOWLEDGEMENTS

This work was funded by a grant from the Alberta Cancer Foundation and by the Alberta Cancer Board Research Initiative Program.

### REFERENCES

Allalunis-Turner MJ, Pearcey RG, Barron GM, Buryn DA, Babiak JC and Honoré LH (1991) Inherent radiosensitivity testing of tumor biopsies obtained from patients with carcinoma of the cervix or endometrium. *Radiother Oncol* **22**: 201–205

Allalunis-Turner MJ, Barron GM, Day RS III, Fulton DS and Urtasun RC (1992) Radiosensitivity testing of human primary brain tumor specimens. *Int J Radiat Oncol Biol Phys* **23**: 339–343

Allalunis-Turner MJ, Lintott LG, Barron GM, Day RS III and Lees-Miller SP (1995) Lack of correlation between DNA-dependent protein kinase activity and tumor cell radiosensitivity. *Cancer Res* **55**: 5200–5202

Britten RA, Evans AJ, Allalunis-Turner MJ and Pearcey RG (1996a) Effect of cisplatin on the clinically relevant radiosensitivity of human cervical carcinoma cell lines. *Int J Radiat Oncol Biol Phys* **34**: 367–374

Britten RA, Evans AJ, Allalunis-Turner MJ, Franko AJ and Pearcey RG (1996*b*) Intratumoral heterogeneity as a confounding factor in clonogenic assays for tumor radiosensitivity. *Radiother Oncol* **39**: 145–153

Bryant PE and Liu N (1994) Responses of radiosensitive repair-proficient cell lines to restriction endonucleases. *Int J Radiat Biol* **66**: 597–601

Cox R, Masson WK, Debenham PG and Webb MBT (1984) The use of recombinant DNA plasmids for the determination of DNA-repair and recombination in cultured mammalian cells. *Br J Cancer* **49** (suppl. 6): 67–72

Deacon J, Peckham MJ and Steel GG (1984) The radioresponsiveness of human tumours and the initial slope of the cell survival curve. *Radiother Oncol* **2**: 317–323

Fairman MP, Johnson AP and Thacker J (1992) Multiple components are involved in the efficient joining of double stranded DNA breaks in human cell extracts. *Nucleic Acids Res* **20**: 4145–4152

Fertil B and Malaise E-P (1981) Inherent cellular radiosensitivity as a basic concept for human tumor radiotherapy. *Int J Radiat Oncol Biol Phys* **7**: 621–629

Ganesh A, North P and Thacker J (1993) Repair and misrepair of site-specific DNA double-strand breaks by human cell extracts. *Mutat Res* **299**: 251–259

Giaccia A, Weinstein R, Hu J and Stomato TD (1985) Cell cycle repair of double-strand DNA breaks in a gamma-ray sensitive Chinese hamster cell. *Somat Cell Mol Genet* **11**: 485–491

Giaccia AJ, Schwartz J, Shieh J and Brown JM (1992) The use of asymmetric-field inversion gel electrophoresis to predict tumor cell radiosensitivity. *Radiother Oncol* **24**: 231–238

Kirchgessner CU, Patil CK, Evans JW, Cuomo CA, Fried LM, Carter T, Oettinger MA and Brown JM (1995) DNA-dependent kinase (p350) as a candidate gene for the murine SCID defect. *Science* **267**: 1178–1183

Johnston PJ and Bryant PE (1994) A component of DNA double-strand break repair is dependent on the spatial orientation of the lesions within the higher-order structures of chromatin. *Int J Radiat Biol* **66**: 531–536

Lambin P, Coco-Martin J, Legal JD, Begg AC, Parmentier C, Joiner MC and Malaise EP (1994) Intrinsic radiosensitivity and chromosome aberration analysis using fluorescence in situ hybridization in cells of two human tumor cell lines. *Radiat Res* **138**: S40–43

Lees-Miller SP, Godbout R, Chan DW, Weinfeld M, Day RS III, Barron GM and Allalunis-Turner J (1995) Absence of p350 subunit of DNA-activated protein kinase from a radiosensitive human cell line. *Science* **267**: 1183–1185

Liu N and Bryant PE (1993) Response of ataxia telangiectasia cells to restriction endonuclease induced DNA double-strand breaks: I cytogenetic characterization. *Mutagenesis* **8**: 503–510

 © Cancer Research Campaign 1997

Luo C-M, Tang W, Mekeel KL, DeFrank JS, Rani Anné P and Powell SN (1996) High frequency and error-prone DNA recombination in ataxia telangiectasia cell lines. *J Biol Chem* **271**: 4497–4503

North P, Ganesh A and Thacker J (1990) The rejoining of double-strand breaks in DNA by human cell extracts. *Nucleic Acids Res* **18**: 6205–6210

Olive PL, Banáth JP and MacPhail HS (1994) Lack of a correlation between radiosensitivity and DNA double-strand break induction or rejoining in six human tumor cell lines. *Cancer Res* **54**: 3939–3946

Olnes MJ and Kurl RN (1994) Isolation of nuclear extracts from fragile cells: a simplified procedure applied to thymocytes. *Bio Techniques* **17**: 828–829

Perrault AR, Cheong N and Iliakis G (1997) Evidence for multiple pathways for DSB rejoining obtained by utilizing plasmid and genomic DNA as substrates. 45th Annual Meeting of the Radiation Research Society, May 3–7 Providence, Rhode Island, USA. P12–252

Powell SN and McMillan TJ (1991) Clonal variation of DNA repair in a human glioma cell line. *Radiother Oncol* **21**: 225–232

Powell SN and McMillan TJ (1994) The repair fidelity of restriction enzyme-induced double strand breaks in plasmid DNA correlates with radioresistance in human tumor cell lines. *Int J Radiat Oncol Biol Phys* **29**: 1035–1040

Powell SN, Whitaker SJ, Edwards SM and McMillan TJ (1992) A DNA repair defect in a radiation-sensitive clone of a human bladder carcinoma cell line. *Br J Cancer* **65**: 798–802

Powell SN, Whitaker S, Peacock J and McMillan TJ (1993) Ataxia telangiectasia: an investigation of the repair defect in the cell line AT5BIVA by plasmid reconstitution. *Mutat Res* **294**: 9–20

Russell NS, Arlett CF, Bartelink H and Begg AC (1995) Use of fluorescence in situ hybridization to determine the relationship between chromosome aberrations and cell survival in eight human fibroblast strains. *Int J Radiat Biol* **68**: 185–196

Sasai K, Evans JW, Kovacs MS and Brown JM (1994) Prediction of human cell radiosensitivity: comparison of clonogenic assay with chromosome aberrations scored with premature chromosome condensation with fluorescence in situ hybridization. *Int J Radiat Oncol Biol Phys* **30**: 1127–1132

Savitsky K, Bar-Shira A, Gilad S, Rotman G, Ziv Y, Vanagaite L, Tagle DA, Smith S, Uziel T, Sfez S, Ashkenazi M, Pecker I, Frydman M, Harnik R, Patanjali SR, Simmons A, Clines GA, Sartiel A, Gatti RA, Chessa L, Sanal O, Lavin MF, Jaspers NGJ, Taylor AMR, Arlett CF, Miki T, Weissman SM, Lovett M, Collins FS and Shiloh Y (1995) A single ataxia telangiectasia gene with a product similar to PI-3 kinase. Science **268**: 1749–1753

Taccioli GE, Gottlieb TM, Blunt T, Priestley A, Demengeot J, Mizuta R, Lehmann AR, Alt FW, Jackson SP and Jeggo PA (1994) Ku80: product of XRCC5 gene and its role in DNA repair and V(D)J recombination. *Science* **265**: 1442–1445

Taylor AMR, Harnden DG, Arlett CF, Harcourt SA, Lehmann AR, Stevens S and Bridges BA (1975) Ataxia telangiectasia: a human mutation with abnormal radiation sensitivity. *Nature* **258**: 427–429

Thacker J and Ganesh AN (1990) DNA-break repair, radioresistance of DNA synthesis, and camptothecin sensitivity in the radiation-sensitive *irs* mutants: comparisons to ataxia-telangiectasia cells. *Mutat Res* **235**: 49–58

Thacker J, Chalk J, Ganesh A and North P (1992) A mechanism for deletion formation in DNA by human cell extracts: the involvement of short sequence repeats. *Nucleic Acids Res* **20**: 6183–6188

West CML, Davidson SE, Roberts SA and Hunter RD (1993) Intrinsic radiosensitivity and prediction of patient response to radiotherapy for carcinoma of the cervix. *Br J Cancer* **68**: 819–823

© Cancer Research Campaign 1997